# Phytosterols in hull-less pumpkin seed oil, rich in ∆^7^-phytosterols, ameliorate benign prostatic hyperplasia by lowing 5α-reductase and regulating balance between cell proliferation and apoptosis in rats

**DOI:** 10.29219/fnr.v65.7537

**Published:** 2021-12-02

**Authors:** Xin-cong Kang, Tian Chen, Jia-li Zhou, Peng-yuan Shen, Si-hui Dai, Chang-qing Gao, Jia-yin Zhang, Xing-yao Xiong, Dong-bo Liu

**Affiliations:** 1 Horticulture College, Hunan Agricultural University, Changsha, Hunan, P. R. China; 2 Hunan Provincial Key Laboratory of Crop Germplasm Innovation and Utilization, Hunan Agricultural University, Changsha, Hunan, P. R. China; 3 State Key Laboratory of Subhealth Intervention Technology, Changsha, Hunan, P. R. China; 4 Hunan Co-Innovation Center for Utilization of Botanical Functional Ingredients, Changsha, Hunan, P. R. China; 5 Department of Laboratory Animals, Xiang Ya Hospital, Central South University, Changsha, Hunan, P. R. China; 6 Institute of Vegetables and Flowers, Chinese Academy of Agricultural Sciences, Beijing, P. R. China; † These authors contributed equally to this work

**Keywords:** lower urinary tract symptoms, 5α-reductase inhibitor, MAPK pathway, apoptosis, proliferation, phytochemicals, Curcurbita pepo

## Abstract

**Background:**

Pumpkin seed oil is widely used to treat benign prostatic hyperplasia (BPH), a common disease in elder men. However, its active components and mechanism have remained to be elucidated.

**Objective:**

The objective of the present study was to investigate the active components of pumpkin seed oil and its mechanism against BPH.

**Design:**

Total phytosterol (TPS) was isolated from hull-less pumpkin (*Cucurbita pepo* L. *var*. *Styriaca*) seed oil and analyzed by gas chromatography/mass spectrometry (GC/MS). Three phytosterols were purified by preparative HPLC (high performance liquid chromatography) and confirmed by NMR (nuclear magnetic resonance). TPS (3.3 mg/kg body weight, 1 mL/day/rat) was administered intragastrically to the testosterone propionate-induced BPH rats for 4 weeks. The structure changes of prostate tissues were assessed by hematoxylin & eosin (H&E) staining. The expression of androgen receptor (AR) and steroid receptor coactivator 1 (SRC-1) was analyzed by immunohistochemistry, while that of 5α-reductase (5AR), apoptosis, or proliferation-related growth factors/proteins was detected by real-time quantitative polymerase chain reaction or western blotting.

**Results:**

The ∆^7^-phytosterols in TPS reached up to 87.64%. Among them, 24β-ethylcholesta-7,22,25-trienol, 24β-ethylcholesta-7,25(27)-dien-3-ol, and ∆^7^-avenasterol were confirmed by NMR. TPS treatment significantly ameliorated the pathological prostate enlargement and restored histopathological alterations of prostate in BPH rats. It effectively suppressed the expressions of 5AR, AR, and coactivator SRC-1. TPS inhibited the expression of proliferation-related growth factor epidermal growth factor, whereas it increased the expressions of apoptosis-related growth factor/gene transforming growth factor-β1. The proliferation-inhibiting effect was achieved by decreasing the ERK (extracellular signal-regulated kinase) phosphorylation, while apoptosis was induced by Caspase 3 activation through JNK (c-Jun N-terminal kinase) and p38 phosphorylation.

**Conclusion:**

TPS from hull-less pumpkin seed oil, with ∆^7^-phytosterols as its main ingredients, is a potential nutraceutical for BPH prevention.

## Popular scientific summary

- ∆7-phytosterol was the main ingredients of total phytosterol (**TPS**), which was isolated from hull-less pumpkin (*Cucurbita pepo* **L**. *var. Styriaca*) seed oil, a well-known functional food.
- **TPS** treatment significantly recovered the symptom of **BPH** in rats by lowering 5α-reductase expression and regulating the balance between cell proliferation and apoptosis.
- As a functional food ingredient, phytosterol might be a promising candidate complementary and alternative medicine for the treatment of **BPH** in the future.

Benign prostatic hyperplasia (BPH) affects ~50% of men aged >50 years and ~90% in their 80s (1). Patients with BPH can exhibit various lower urinary tract symptoms (LUTSs), including hesitancy, incomplete voiding, post-void dribbling, or irritative symptoms (2). It is characterized by hyperplasia of the mesenchymal stromal and glandular epithelial cells in the prostate (3).

Although the pathophysiological mechanism of BPH still needs to be investigated, the predominant hypothesis is age-dependent alterations in hormone ratio and age-related tissue remodeling (4). Testosterone and dihydrotestosterone (DHT) are considered to be related to the development of BPH. By the action of 5α-reductase (5AR), testosterone is converted into DHT, a more potent androgen with higher affinity for the androgen receptor (AR). Serum concentration of testosterone decreases with age, whereas the activities of 5AR and AR are increased due to androgen balance. Recent studies suggest that the development of BPH involves the disruption of the DHT-supported homeostasis between cell proliferation and cell death, allowing proliferative processes to predominate (5). The mitogen-activated protein kinase (MAPK) signaling pathways, linking surface receptor-mediated signals to nuclear events, play key roles in the cellular proliferation, growth, and programmed cell apoptosis (6). Because of the different cell type and different stimulus, the activation of a MAPK signaling pathway might lead to opposite effect (7).

As one of the most frequently prescribed medications for BPH patients, the 5AR inhibitor can block the conversion of testosterone into DHT. Finasteride (Fi), a representative drug for BPH treatment, is one of 5AR inhibitors. It not only improves urinary flow rate but also reduces the risk of acute urinary retention and the requirement for surgical intervention. However, the sexual side effects, including decreased libido, reduced ejaculate volume, and erectile dysfunction, are significantly bothersome (8). Phytotherapeutic agents have recently gained interest worldwide due to their advantages of nature and harmlessness (9).

Pumpkin (*Cucurbita* spp.) has received considerable attention because of the nutritional and health-protective value of its seed oil. Pumpkin seed oil has been reported to be effective in improving BPH and other LUTS (10, 11). It contains a wide variety of bioactive compounds, including fatty acids, phytosterols, vitamins, etc. (12–14). However, the active ingredients and their mechanisms against BPH are unclear. Phytosterol, especially beta-sitosterol, is routinely suggested as the major active component against BPH (15–17). However, Tsai et al. considered that phytosterol produced a limited effect (18).

In this study, we isolated the total phytosterols (TPSs) of hull-less pumpkin seed oil and analyzed their composition. The hull-less seed pumpkin is a spontaneous mutant of standard pumpkin (*Cucurbita pepo* L. *var*. *Styriaca*), losing hard husk. Its seeds could be processed conveniently. To investigate the therapeutic potential of TPS against BPH, we observed the histopathological change of prostate in a male Sprague–Dawley (SD) rat model. Furthermore, to explore the molecular mechanism behind the effect, we evaluated the mRNA (messenger RNA) and/or protein expression of 5AR, AR, and coactivator SRC-1 (steroid receptor coactivator 1). Growth factors and related proteins in cell apoptosis and proliferation were also detected, including Caspase 3, JNK, ERK, p38, etc.

## Materials and methods

### Preparation of TPS in hull-less pumpkin seed oil

The seeds of hull-less pumpkin (*Cucurbita pepo* L. *var*. *Styriaca*), which was identified by Prof. S.H. Dai (Hunan Agricultural University, Changsha, Hunan, China), were provided by Hunan provincial engineering research center for Cucurbitaceae. A voucher specimen (CPS-201710) was deposited at the Hunan provincial engineering research center for Cucurbitaceae. The pumpkin seed oil was extracted by supercritical carbon dioxide (CO^2^) fluid and then incubated in ethanolic potassium hydroxide. After extracted with an equal volume of n-hexane, the n-hexane solution was washed successively with 30% volume of ddH^2^O, 20% ethanol, ddH^2^O, 0.5 mol/L potassium hydroxide, ddH^2^O, until the n-hexane phase was clear. The light n-hexane was dehydrated by moderate anhydrous sodium sulfate. Afterward, the TPS was obtained by concentration, freeze-crystallization, aspirator filter, and drying.

### Gas chromatography–mass spectrometry analysis, compounds isolation, and validation

The composition of TPS was analyzed using gas chromatography–mass spectrometry (GC-MS; Shimazu GCMS-QP 2020, Tokyo, Japan), equipped with an electrospray ionization (EI) source and a Rtx^®^-5MS Capillary column (30 m × 0.25 mm × 0.25 μm). TPS was silylated derived with BSTFA+TMCS (Bis(trimethylsilyl)trifluoroacetamide+Trimethylchlorosilane 99:1) at 80°C for 40 min. After cooled to room temperature, C^2^Cl^2^ was added for dissolving. The peaks were then tentatively identified from their retention characteristics and mass fragmentation patterns after initial pretreatment by using NIST.14 mass spectrum database. Three of the peaks were isolated by preparative high-performance liquid chromatography (HPLC) and were validated by NMR. 1H-NMR (400 MHz) and 13C-NMR (100 MHz) spectra were obtained at 25°C with CDCl^3^ as solvent on a Bruker AVANCE 500 M NMR instrument (Bruker, Switzerland).

### Animals

The 12-week-old male SD rats (*n* = 40) weighing 160–200 g were purchased from Hunan Slake Jingda Experimental Animal Co. Ltd (No. 42004700048405). The experimental protocol was approved by the research Ethics Committee of the Third Xiangya Hospital, Central South University (SCXK [Xiang] 2016-0002). The rats were housed in a pathogen-free room maintained at a temperature of 23 ± 1°C and relative humidity of 70% with an alternating 12 h light/dark cycle. Water and standard laboratory diet were provided *ad libitum*. They were allowed for acclimation under climate-controlled conditions for 7 days before the experiments began.

### Experimental design

All rats were randomly divided into four groups with 10 animals in each group, including 1) a normal control (NC) group, 2) BPH group, 3) Fi group: a positive control group, BPH + 1 mg/kg Fi, and 4) TPS group: BPH + 3.3 mg/kg TPS. TPS was dissolved in corn oil and orally administered to the mice (1 mL/day/rat), while the NC group were treated with the vehicle. To eliminate the influence of endogenous testosterone, rats in all groups except the NC group underwent bilateral orchiectomy 3 days prior to testosterone treatment. For the orchiectomy, the animals were anesthetized by intraperitoneal injections of sodium pentobarbital (25 mg/kg body weight, BW). The rats in BPH-induced groups were castrated and then induced by a pre-4-week treatment of daily subcutaneous injections of testosterone propionate (5 mg/kg) at the inguinal region (*n* = 30). TPS and Fi were intragastric administration once a day for 4 weeks. At the end of the experimental period, all rats were sacrificed by sodium pentobarbital anesthetization (25 mg/kg BW, *i.p.*) followed by cervical dislocation. The prostates were removed from all rats. Each prostate was weighed and divided into three parts. One was fixed in 10% formalin and embedded in paraffin for histomorphological analysis, and the others were stored at −80°C for further assays.

### Hematoxylin & eosin staining and immunohistochemistry

The structure changes of prostate tissues, including the epithelial thickness and lumen area, were assessed by hematoxylin & eosin (H&E) staining. AR and SRC-1 were analyzed by immunohistochemistry (IHC), which was performed on formalin-fixed, paraffin-embedded tissue sections from archival blocks using standardized avidin-biotin techniques. Antibodies for AR and SRC-1 (dilution of 1:100) were purchased from Abcam, Inc. (Cambridge, United States) and Sigma-Aldrich Inc. (St. Louis, MO, USA), respectively.

### Real-time quantitative polymerase chain reaction analysis and western blotting assay

The mRNA expression was analyzed by real-time quantitative polymerase chain reaction (RT-qPCR) and calculated by Livak 2^-ΔΔCt^ method (19). Beta-actin was used as the house-keeping gene. All primers were designed by Beacon Designer 7.0 (PREMIER Biosoft International, San Francisco, USA) and synthesized by TsingKe Biological Technology Co. Ltd. (Changsha, China). Protein expressions were analyzed by western blotting. Total proteins extracted from liver tissue lysates were separated by 10% SDS-PAGE and transferred onto polyvinylidene difluoride (PVDF) membranes. Then, PVDF membranes were blocked with skimmed milk (5% in PBS containing 0.2% Tween-20) at room temperature for 2 h and probed with primary antibodies overnight at 4°C. Antibodies for Caspase 3/Cleaved-Caspase 3, ERK/p-ERK, JNK/p-JNK, and p38/p-p38 were purchased from Abcam, Inc. (Cambridge, United States). Membranes were washed and labeled with secondary antibodies of goat anti-mouse IgG1 (Southern Biotech, USA) for 2 h at room temperature. The protein bands were visualized using ECL Prime Western Blotting Detection Reagent (Bio-Rad, USA). The chemiluminescent intensities of protein signals were quantified using Image J v1.8.0 software (National Institutes of Health, USA).

### Statistical analysis

The results were expressed as mean ± standard deviation (SD). Comparison between groups was performed using one-way analysis of variance. Statistical significance was accepted at *P* < 0.05.

## Results

### Chemical characterization of TPS in hull-less pumpkin seed oil

**Fig. 1:**
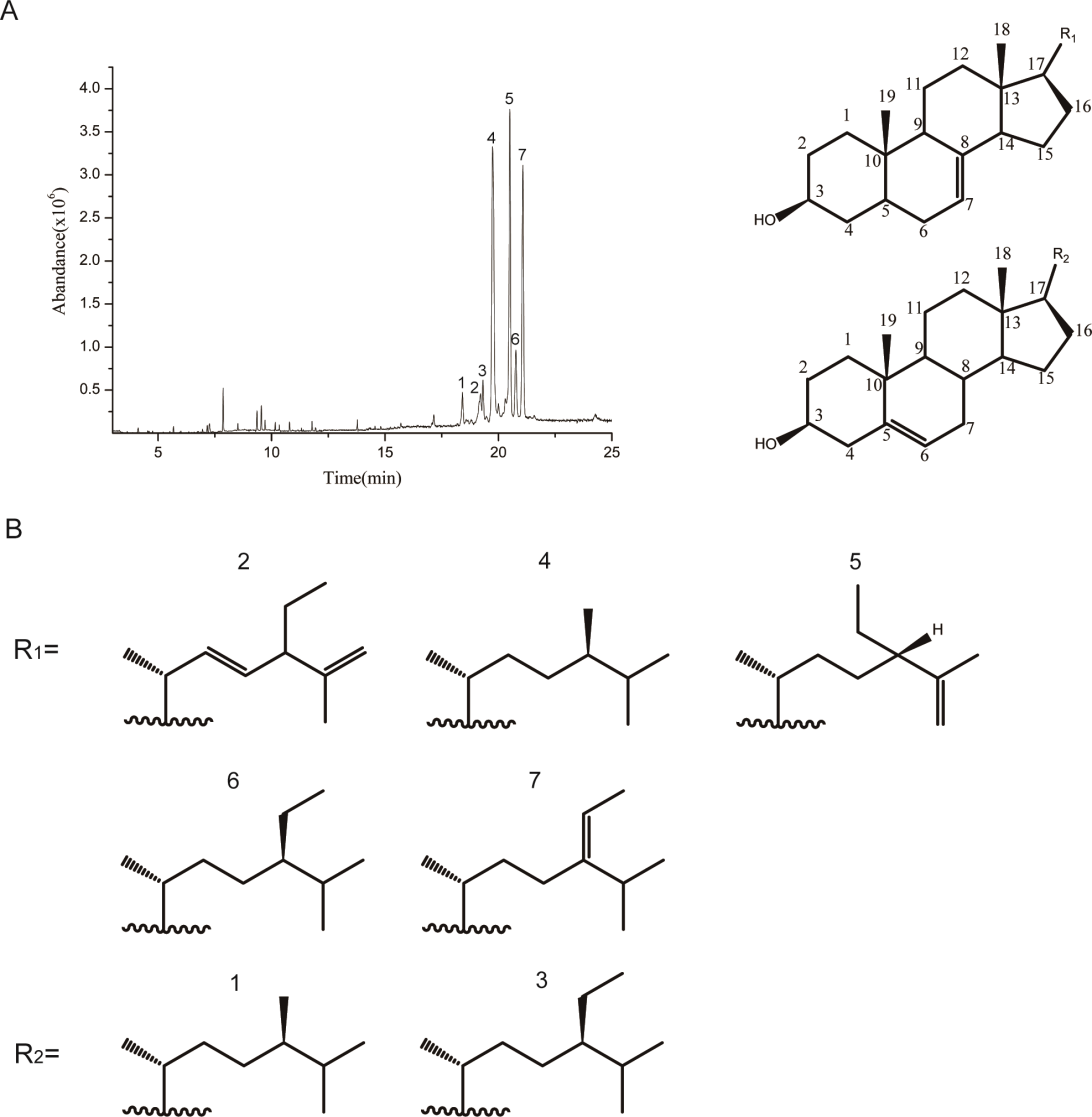
GC–MS analysis of TPS in hull-less pumpkin seed oil. (A) Total ion chromatogram of TPS by GC–MS. (B) Chemical structure of peaks (1–7) shown in (A). Peaks (1–7) were identified by comparing with NIST.14 standard library and defined in the text.

As shown in Fig. 1 and Table 1, the hull-less pumpkin seed oil was rich in phytosterols. The TPS compounds found in the oil included 1) campeterol, 2) 24β-ethylcholesta-7, 22, 25-trienol, 3) β-sitosterol, 4) ∆^7^-campeterol, 5) 24β-ethylcholesta-7, 25(27)-dien-3-ol, 6) ∆^7^-sitosterol, and 7) ∆^7^-avenasterol (Fig. 1 and Table 1). In contrast to the other vegetable oils, in which ∆^5^-phytosterols are the main ingredients, the TPS contained 87.64% of ∆^7^-phytosterols. We obtained compounds 2, 5, and 7 with a purity of 90, 94.6, and 91.3%, respectively, using preparative HPLC and validated by NMR. The physical, analytical, and spectral data of compounds 2, 5, and 7 were shown in the Supplementary material and identified as (2) 24β-ethylcholesta-7, 22, 25-trienol, (5) 24β-ethylcholesta-7, 25(27)-dien-3-ol, and (7) ∆7-avenasterol, according to the references (20, 21).

**Table 1:** Compounds of TPS in hull-less pumpkin seed oil

Peak	Compound	A%
1	Campeterol	3.75
2	24β-Ethylcholesta-7,22,25-trienol	2.41
3	β-Sitosterol	8.17
4	∆^7^-Campeterol	23.56
5	24β-Ethylcholesta-7,25(27)-dien-3-ol	30.75
6	∆^7^-Sitosterol	12.59
7	∆^7^-Avenasterol	18.33

Note: Peaks 1–7 are the peaks 1–7 in Fig. 1A.

### Effect of TPS on histopathologic patterns

**Fig. 2:**
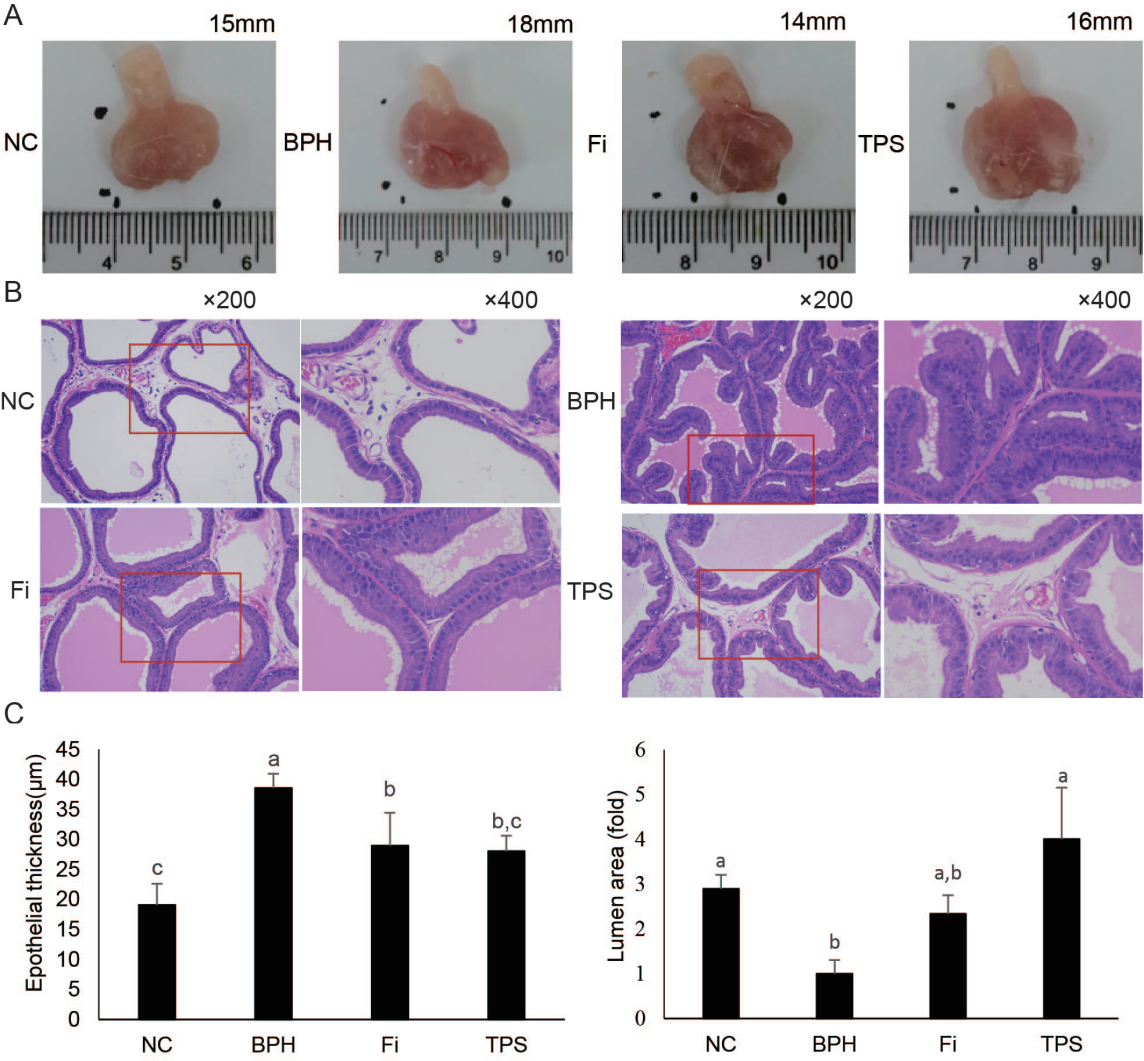
The effect of TPS on histopathologic patterns of prostate tissue. (A) Size of prostate tissue; (B) representative photomicrograph of H&E-stained prostate tissues (left panel magnification ×200 and right panel magnification ×400); (C) epithelial thickness and lumen area level. Data are means ± SD (*n* = 6 for each group). Different lowercase letters (a–c) show a significant difference between groups (*P* < 0.05).

No significant difference was observed in the average BW of rats from all groups (Table 2). The average prostate size, prostate weight (PW), and prostate weight index (PWI) of the BPH group were significantly higher than those of the NC group (Fig. 2A and Table 2). The increment of PW of the TPS group was significantly lowered by 83.4% than those of the BPH group (Table 2). The effect was comparable as that of Fi’s. Similar results were observed in TPS group’s PWI, which was inhibited by 65.8% (Table 2).

**Table 2:** Effect of TPS on prostatic parameters

Group	Body weight (g)	PW (mg)	PWI (mg/100 g)	Inhibition of increase in PWI (%)
NC	450.4 ± 47.7	835.6 ± 161.9^b^	185.6 ± 29.5^b^	–
BPH	415.8 ± 25.1	1072.9 ± 61.9^a^	258.8 ± 21.0^a^	–
Fi	439.2 ± 32.7	868.4 ± 188.3^b^	198.1 ± 44.3^b^	82.9
TPS	421.2 ± 48.9	875.1 ± 132.2^b^	210.6 ± 41.1^b^	65.8

Prostate weight index = the prostate weight (mg)/body weight of the rat (100 g).

Inhibition of increase in PWI (%) = (PWI of BPH group – PWI of TPS group)/(PWI of BPH group – PWI of NC group).

Values are mean ± SD (*n* = 10 for each group).

Different lowercase letters (a, b) show a significant difference between groups (*P* < 0.05).

The tubular glands of the BPH group demonstrated typical features of glandular hypertrophy, including thickening of the prostatic epithelial layer, papillary fronds protruding into the glandular cavities, and decreased luminal volume (Fig. 2B). Administration of TPS decreased the prostatic epithelial thickness and increased the glandular luminal area (*P* < 0.05, Fig. 2B and C).

## Effect of TPS on 5AR, AR and SRC-1 expression

**Fig. 3:**
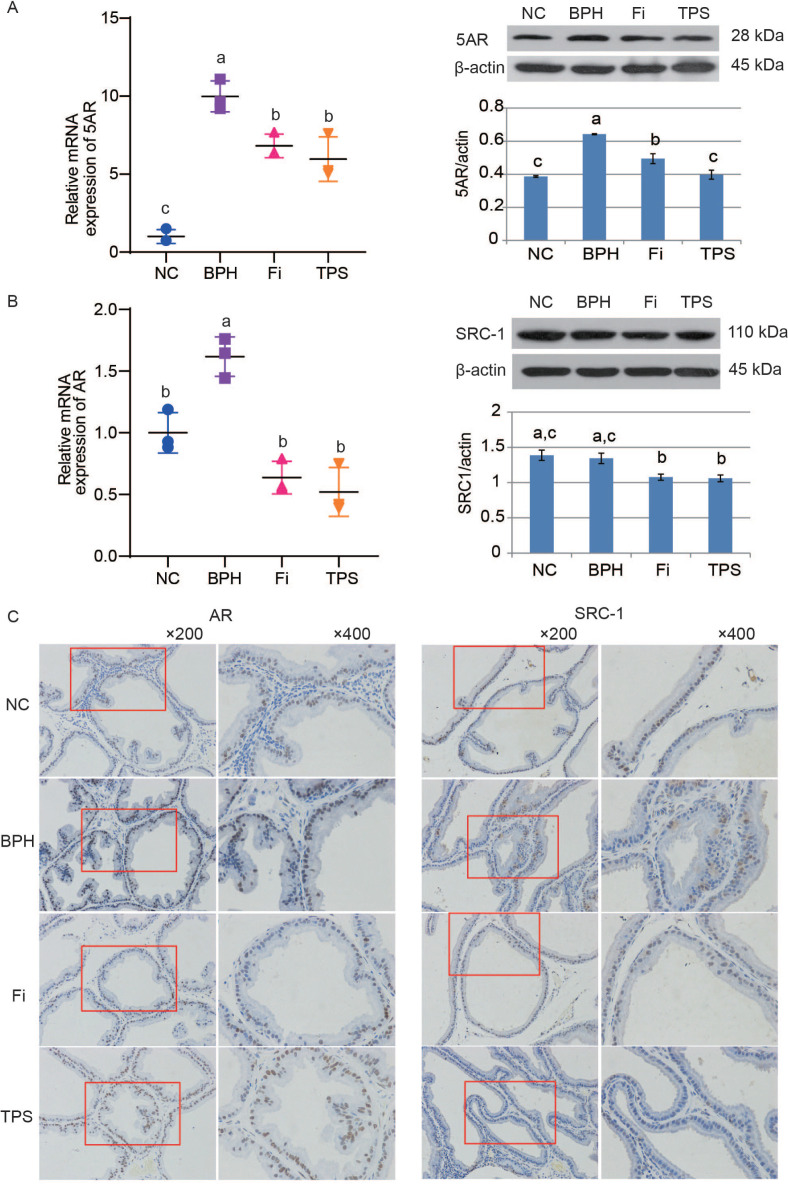
The effect of TPS on 5AR, AR, and its coactivator SRC-1 expression of the prostate tissues in testosterone propionate-induced BPH rats. (A) Relative mRNA expression and protein expression of 5AR in prostate tissues; (B) relative mRNA expression of AR and protein expression of SRC-1; (C) representative photomicrograph of IHC-stained prostate tissues with anti-AR and anti-SRC-1 antibodies (left panel magnification ×200 and right panel magnification ×400). Data are mean ± SD from three independent experiments. Different lowercase letters (a–c) show a significant difference between groups (*P* < 0.05).

Testosterone propionate enhanced the 5AR expression in both mRNA and protein levels in the BPH group (Fig. 3A). Treatment with TPS was demonstrated to reduce the testosterone propionate-induced 5AR expression alteration (Fig. 3A). RT-qPCR results showed that the mRNA expression of AR was upregulated in the BPH group by comparing with those of the NC group. TPS reduced the elevated AR mRNA expression in rats (Fig. 3B). The immunofluorescence staining of AR protein confirmed the RT-qPCR results (Fig. 3B and C). Although the protein expression of SRC-1, a typical type of AR adjuvant, showed no significant difference in the BPH group by western blotting, it showed a reduction by TPS treatment, both in the western blotting and IHC assay (Fig. 3B and C).

### Effect of TPS on cell proliferation and apoptosis

**Fig. 4:**
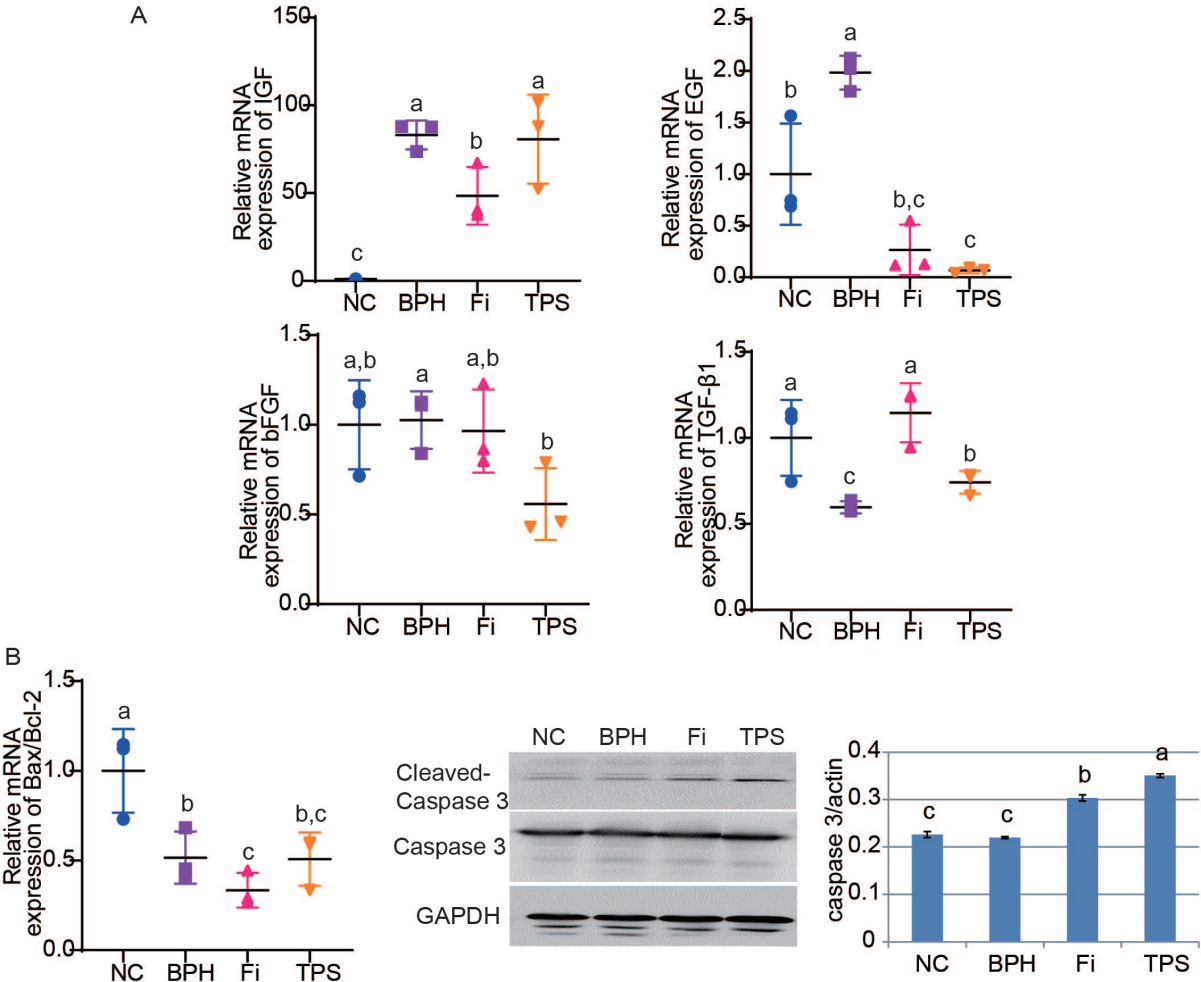
The effect of TPS on cell proliferation and apoptosis in testosterone propionate-induced BPH rats. (A) Relative mRNA expression of proliferation-related growth factors IGF, EGF, bFGF, and TGF-β1; (B) relative Bax/Bcl-2 mRNA expression and Cleaved-Caspase 3 protein expression. Data are means ± SD from three independent experiments. Different lowercase letters (a–c) show a significant difference between groups (*P* < 0.05).

After treated with testosterone propionate, the mRNA relative expressions of growth factors insulin-like growth factor (IGF) and epidermal growth factor (EGF) were markedly increased (Fig. 4A). With the administration of TPS, the mRNA relative expressions of EGF significantly decreased, whereas those of IGF showed no significant difference (Fig. 4A). The basic fibroblast growth factor (bFGF) expression showed no significant difference in the BPH group, while it was decreased in the TPS group (Fig. 4A). For the proapoptotic gene transforming growth factor-β1 (TGF-β1), it was downregulated in the BPH group and upregulated after treating with TPS (Fig. 4A). The expression of the apoptosis-effector, cleaved-Caspase 3, was elevated after treated with TPS in the western blotting; however, the Bax/Bcl-2 mRNA expression showed no significant difference (Fig. 4B).

### Effect of TPS on MAPK signal expression

**Fig. 5:**
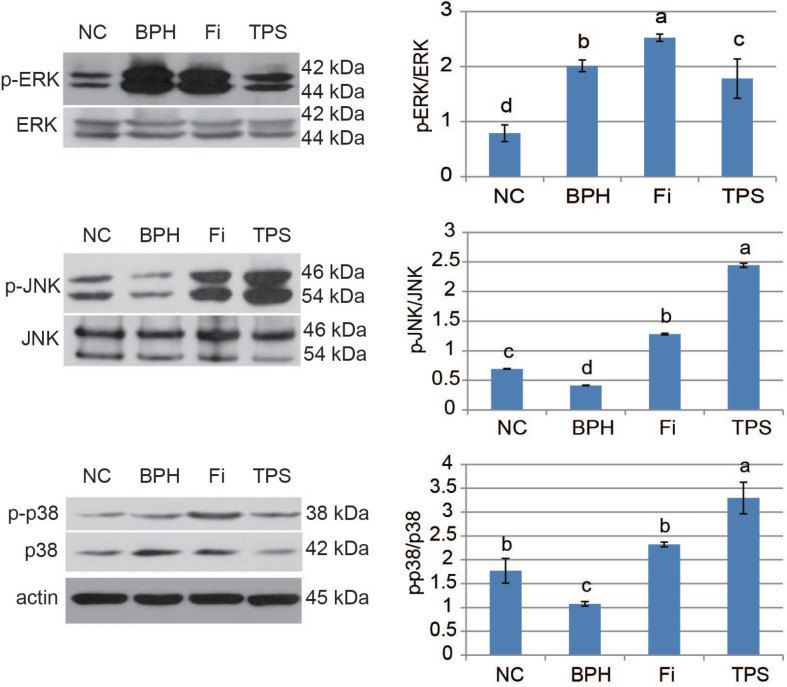
The effect of TPS on MAPK signal expressions in prostate tissues of testosterone propionate-induced BPH rat. Phosphorylation levels of ERK, JNK, and p38 were analyzed by western blotting. Data are means ± SD from three independent experiments. Different lowercase letters (a–d) show a significant difference between groups (*P* < 0.05).

Induced by testosterone propionate, the expression levels of p-ERK were increased obviously (Fig. 5). After the management of TPS, the expression level of p-ERK in the TPS group was significantly reduced (Fig. 5). For p-JNK and p-p38, it showed reduction in the BPH group and increase in the Fi and TPS groups (Fig. 5).

## Discussion

In this study, TPS, composed of ∆^7^-phytosterols and ∆^5^-phytosterols, was isolated from hull-less pumpkin seed oil. It showed admirably inhibited effects on BPH. Beta-sitosterol, one of ∆^5^-phytosterols, has been reported to improve the symptoms and urinary flow parameters of BPH (22). Beta-sitosterol reduced the conversion of testosterone to DHT and inhibited proliferation of human prostate cancer cells (23, 24). In contrast, Tsai et al. fed rats with pumpkin seed oil combined with ∆^5^-phytosterol and found that ∆^5^-phytosterol (β-sitosterol, campesterol, stigmasterol, and brassicasterol) produced a limited additive effect of pumpkin seed oil on the BPH (18). In this study, β-sitosterol only accounts for 8.17% of TPS, and ∆^7^-phytosterols reached up to 87.64%. Thus, we supposed that ∆^7^-phytosterols might be the active ingredients for BPH treatment.

DHT overproduction could lead to the development and exacerbation of BPH. Numerous researchers have aimed to reduce DHT levels by inhibiting 5AR (25). The 5AR is responsible for the synthesis of DHT, which has substantially greater affinity for AR than testosterone does. DHT could induce the overexpression of AR, which promotes BPH development via enhancing the recruitment of infiltrating macrophages that resulted in increased stromal cell proliferation (26). DHT binds to an AR and SRC1 and forms a complex, which shows high affinity to specific androgen response elements. It causes the production of prostate-specific antigen and regulatory proteins that modulate cell proliferation and death (5). As reported, the positive agent Fi showed a significant inhibition of 5AR, AR, and coactivator SRC-1 expression, resulting in reduction of prostate size and improvement of LUTS (27, 28). In this study, TPS showed similar therapeutic potential against BPH as Fi did.

An imbalance between cell proliferation and cell death leads to the abnormal growth of prostate cells, the major pathological feature of BPH (29, 30). Three MAPK cascades were implicated in these pathological alterations by modulating the local prostatic environment to favor the progression of BPH. It is known that MAPK family members participate in regulating the cell cycle in various ways (31). ERK mainly is involved in regulating the progression of G0/G1 to the S phase, and p38 primarily regulates the G2 checkpoint (32, 33). ERK pathway is reported to be essential for the proliferation of human prostate (28, 34). Its activation might result in mitogenesis and induces motility (35). JNK-dependent apoptosis was suppressed by the activation of ERK MAPK (36). JNK not only induce the Bax phosphorylation and mitochondrial translocation to promote apoptosis but also phosphorylate and inactivate Bcl-2 to suppress its antiapoptotic function. JNK and p38 always work in tune, and the activation and role of p38 seem to be similar to the JNK pathway (7). In this study, there is a dominance of ERK cascade and suppression of JNK and p38 cascades in BPH, which is agreed with the notion of Athanasios et al. (6). TPS could inhibit the ERK cascade and activate the JNK and p38 cascades, leading to the repression of proliferation and activation of apoptosis. However, in the apoptosis-promoting process, TPS could not increase the expression of downregulated Bax/Bcl-2. The down-effector of JNK and p38 after TPS administration needs further study.

Growth factors EGF, bFGF, IGF, and TGF-β are activators of MAPK signaling (37–39). The production and secretion of EGF and IGF may be stimulated by DHT (5). In prostate, the activation of EGF receptor and IGF receptor leads to ERK/MAPK activation (35). In benign and malignant prostate, the overexpression of EGF, bFGF, and IGF protein has been observed (37, 40, 41). EGF is a mitogen for prostatic epithelial cells and has been proven to promote the growth of prostate (42). Additionally, EGF can greatly enhance the expression of VEGF in BPH (43). The strong EGF-induced responses of BPH could contribute to the transformation/malignant potential (44). The activity of TGF-β may be affected by DHT (5). TGF-β has an inhibitory role in BPH, as well as in the normal prostate, inhibiting proliferation and inducing apoptosis in epithelial cells (45, 46). Although the activation mechanisms and its biological consequences of TGF-β poorly characterized, it is known that it can activate the ERK, JNK, and p38 MAPK kinase pathways (47). In this study, TPS inhibited BPH development by decreased the mRNA expression of EGF and increased the expression of TGF-β.

**Fig. 6:**
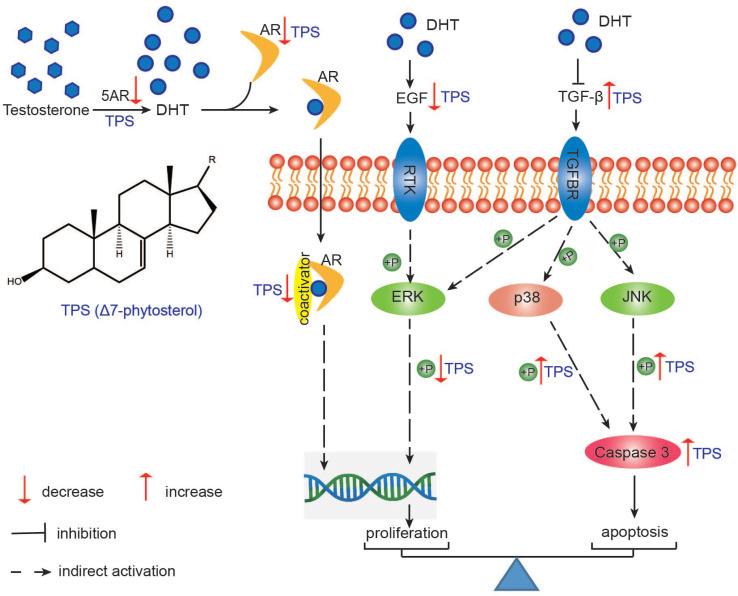
The proposed mechanism of TPS ameliorating BPH in testosterone propionate-induced BPH rat. This graph is a summary of the mechanism speculated from this study. TPS: total phytosterols; 5AR: 5α-reductase; AR: androgen receptor; TGFBR: TGF-β receptor; RTK: receptor tyrosine kinase.

In conclusion, TPS from hull-less pumpkin seed oil, with ∆^7^-phytosterols as the main ingredients, exhibited anti-BPH effect *via* lowering the 5AR expression, regulating the balance between proliferation and apoptosis. The proliferation-inhibited action was conducted by lowering the AR and SRC-1 expressions and decreasing the phosphorylation of ERK. The apoptosis was promoted by Caspase 3 activation through JNK and p38 phosphorylation (Fig. 6). This study indicated that phytosterols of hull-less pumpkin seed oil could be a potential nutraceutical for the prevention and treatment of BPH.
